# Supplementary material for: Effects of interactivity, immersion, and physical discomfort on learning in VR nursing education
Source: PLoS One. 2026 Mar 11;21(3):e0344586. doi: 10.1371/journal.pone.0344586 (PMC12978480; doi:10.1371/journal.pone.0344586)
Supplement: S1 Appendix — This appendix provides the full procedural steps, interaction modes (controller manipulation and gaze-based selection), standardized cueing system, and corrective feedback mechanisms used in the VR nursing training modules. (DOCX) [file pone.0344586.s001.docx]

**S1 Appendix**

The design of each teaching module strictly adhered to standardized nursing protocols and followed a fixed procedural sequence to ensure consistent scenario complexity across all participants. Two interaction modes were provided: handheld controller manipulation and gaze-based selection mechanisms.

A unified cueing system was implemented to standardize instructional guidance and minimize unnecessary procedural confusion. Specifically, yellow highlights indicated interactable objects, blue highlights marked the currently selected item, and instructional cues—consisting of brief text messages and directional arrows—guided users to the appropriate action or location.

In addition, the system incorporated an immediate corrective error-feedback mechanism. When an incorrect operation was performed, a red visual indicator was instantly triggered to signal a corrective warning. Users were required to rectify the error before proceeding to the next stage of the task.

This study included three VR-based skills training modules:

- **Module A:** Meal preparation, feeding, and medication assistance
- **Module B:** Heimlich manoeuvre
- **Module C:** Vital sign measurement

The following sections describe the detailed design of each module.

**Module A: Meal preparation, feeding, and medication assistance**

An 80-year-old male older adult with dysphagia is receiving enteral feeding via a nasogastric (NG) tube. The training scenario covers the complete care process, including preparation, cooking, feeding, communication, patient education, nasogastric tube medication administration, cleaning, and documentation.

**Task Procedures**

**(A) Pre-meal Preparation**

1. Removal of personal accessories
2. Seven-step hand hygiene procedure (hand-motion detection enabled)
3. Preparation of required equipment and materials

**(B) Meal Preparation Process**

1. Cleaning virtual utensils and food ingredients
2. Performing dicing actions and placing prepared ingredients on designated plates
3. Adding ingredients and rice into the container to simulate porridge preparation
4. Performing a stirring action
5. Performing a tasting action
6. Serving the porridge by filling the bowl to approximately 80% capacity and transferring it to the dining table

**(C) Feeding Process**

1. Communicating with the older adult and applying a protective bib
2. Testing food temperature using the inner wrist (symbolic action)
3. Feeding five spoonfuls of porridge, with verbal interaction required for each spoonful
4. Assisting with oral hygiene after feeding

**(D) Nasogastric Tube Medication Administration Process**

1. Explaining medications and confirming the patient’s condition
2. Crushing and mixing medications appropriately
3. Kinking the nasogastric tube and aspirating to confirm tube placement
4. Administering the medication solution in divided doses
5. Flushing the nasogastric tube
6. Documenting care-related information

**Module B: Heimlich Manoeuvre**

This module simulates an older adult experiencing choking while seated at a dining table during a meal. The VR system emphasizes accurate position selection and symbolic action detection rather than real thrust force.

**Task Procedures**

1. Assessing the choking situation
2. Selecting the emergency call option and assisting the older adult to the designated position
3. Selecting correct hand placement and performing a symbolic inward–upward abdominal thrust
4. System feedback indicating foreign body expulsion

**Module C: Vital Sign Measurement**

This module focuses on procedural training for vital sign assessment in an older adult.

**Task Procedures**

1. Confirming the patient’s identity
2. Selecting and preparing the appropriate vital sign measurement equipment
3. Measuring body temperature using the designated device (symbolic action)
4. Measuring blood pressure by selecting correct cuff placement and initiating the measurement sequence
5. Measuring pulse rate through correct site selection and action confirmation
6. Measuring respiratory rate through observation-based symbolic input
7. Confirming and recording vital sign results
