# Supplementary material for: Effects of interactivity, immersion, and physical discomfort on learning in VR nursing education
Source: PLoS One. 2026 Mar 11;21(3):e0344586. doi: 10.1371/journal.pone.0344586 (PMC12978480; doi:10.1371/journal.pone.0344586)
Supplement: S2 Appendix — This appendix contains all measurement items for immersion, interactivity, intrinsic motivation, situational interest, embodied learning, self-efficacy, extraneous cognitive load, and physical discomfort. (DOCX) [file pone.0344586.s002.docx]

**S2 Appendix**

Immersion

1. I felt as if I actually existed in the VR environment.
2. I felt as though I was personally participating in the virtual caregiving activities.
3. I felt as if I had transitioned from the real world to the VR world.
4. I experienced the sensation of being in a virtual reality environment, as if I were truly there.

Interactivity

1. I felt that the VR environment gave me the sense that I could interact with it.
2. I felt that I was able to actively participate in activities within the VR environment.
3. I felt that I could freely move around within the VR environment.
4. I felt that I could accomplish various tasks I wanted to do in the VR environment.

Intrinsic Motivation

1. I really enjoy using virtual reality to simulate caregiving work
2. Using virtual reality to simulate caregiving is fun
3. Performing care work in virtual reality is very interesting
4. Virtual reality doesn't catch my attention at all (reverse scored)

Situational Interest

1. I will enjoy today's assigned task scenarios
2. I think today's assigned tasks are interesting
3. I expect to grasp the theoretical foundations learned today
4. I'm fully focused on today's tasks: I'm not distracted by other things

Embodied learning

1. My physical movements were essential to the learning process in the VR lesson.
2. I used movements and interactions that helped me learn during the VR lesson.
3. Gestures and movements in the VR environment improved my learning experience

Self-Efficacy

1. I'm confident I can understand the basic concepts of long-term care
2. I believe I understand complex concepts related to long-term care
3. I believe I can perform well on the tasks just tested
4. I expect to perform well in long-term care

Extraneous Cognitive Load

1. This task requires remembering many things simultaneously
2. I find this task operation very complex
3. I've done my best since this task requires understanding many details and background.

Head discomfort

1. I experienced a headache during or after VR use.
2. I felt dizzy/vertiginous during or after VR use.
3. I experienced nausea or migraine-like symptoms during or after VR use.

Limb pain

1. I felt pain or stiffness in my fingers while using VR controllers.
2. I felt pain or stiffness in my wrists during or after VR use.
3. I experienced forearm or elbow pain/stiffness during or after VR use.
4. I experienced upper-arm soreness or fatigue during or after VR use.
5. After standing/moving in VR, my legs felt sore or fatigued.

NSB pain

1. I felt neck pain or stiffness during or after VR use.
2. I felt shoulder pain or stiffness during or after VR use.
3. I felt back pain or stiffness during or after VR use.

Visual discomfort

1. I felt eye pain during or after VR use.
2. I experienced dryness or burning in my eyes during or after VR use.
3. I experienced blurred vision during or after VR use.
4. My eyes felt tired during or after VR use.
5. I experienced eye irritation/itching during or after VR use.
6. I experienced light sensitivity (photophobia) during or after VR use.
