# Supplementary material for: Effects of interactivity, immersion, and physical discomfort on learning in VR nursing education
Source: PLoS One. 2026 Mar 11;21(3):e0344586. doi: 10.1371/journal.pone.0344586 (PMC12978480; doi:10.1371/journal.pone.0344586)
Supplement: Attachment — Submitted filename: Response to Reviewers.docx [file pone.0344586.s004.docx]

We sincerely thank the Editor and all three reviewers for their careful evaluation of our manuscript and for the constructive suggestions provided. We have revised the manuscript accordingly and believe that the revisions have substantially strengthened the clarity, rigor, and transparency of the study. Below, we provide point-by-point responses to each comment. All changes referenced below correspond to the revised manuscript.

Reviewer #1:

1. The finding that neck–shoulder–back (NSB) pain increases immersion is unusual and contradicts most prior research. However, the manuscript only provides a brief speculative explanation.

Response:

Thank you for pointing this out. In response, we have expanded the Discussion section to provide additional interpretation and to emphasize the associative nature of this finding. The revised text is provided below and has been added to the manuscript (Lines 436-448).

“A central and unexpected finding was the positive association between NSB pain and immersion. While earlier research has generally shown that musculoskeletal discomfort detracts from VR experience [60, 41], two explanations may account for the current pattern. First, procedural nursing tasks require sustained concentration and repeated upper-body movements. Learners who were more engaged may have simultaneously reported higher immersion and greater musculoskeletal strain. In such cases, NSB pain may reflect the bodily load associated with high task engagement rather than contributing directly to immersive experience. Second, prior research suggests that sensorimotor involvement and bodily feedback can shape users’ subjective experience in virtual environments [17]. From this perspective, musculoskeletal sensations may be integrated into the action–perception cycle during VR training, thereby co-occurring with heightened immersion. These interpretations remain associative rather than causal, but they highlight the nuanced ways in which physical sensations may accompany immersive learning experiences in action-oriented VR tasks.”

2. The Methods section briefly states that the VR modules covered three nursing skills using Unity and Autodesk 3DS Max, but does not describe scenario difficulty, feedback design, or instructional cues.

Response:

Thank you for this valuable comment. In response, we have expanded the Methods section to provide a concise description of how scenario complexity was controlled, how feedback was delivered, and how instructional cues were standardized across the three VR nursing modules. To maintain clarity in the main text, detailed procedural steps, interaction rules, and cueing and feedback specifications are provided in Appendix A. The revised text inserted into the Methods section is shown below (Lines 293-298).

“To enhance reproducibility and maintain consistency across participants, each module employed a standardized sequence of actions, fixed scenario complexity, and a unified cueing system (e.g., on-screen prompts and color-change indicators). Immediate corrective feedback was provided when required procedural steps were missed or performed incorrectly. Full procedural steps, interaction rules, and cueing/feedback specifications are documented in Appendix A”

3. provide more detailed descriptions of scenario complexity, interactivity levels, feedback mechanisms, and how consistency across participants was ensured. This information is necessary for reproducibility.

Response:

Thank you for this suggestion. We have added a concise methodological description in the Methods section specifying standardized action sequences, fixed scenario complexity, unified cueing, and immediate corrective feedback. Full procedural steps and interaction rules are provided in Appendix A.

4. The paper employs PLS-SEM with a cross-sectional survey and a single VR session.
Clarify the justification for using PLS-SEM to test directional hypotheses, especially where causal language is used. Consider explicitly acknowledging limitations due to cross-sectional design and discuss how future longitudinal work could better validate the proposed causal mechanisms.

Response:

Thank you for requesting clarification regarding the use of PLS-SEM. PLS-SEM was selected because it is well suited for models involving multiple latent psychological constructs, does not require multivariate normality, and performs robustly with moderate sample sizes. Importantly, the specified path directions reflect theoretical expectations, not causal claims.

In response, we have revised all hypothesis statements and related descriptions to use predictive or associative terminology (e.g., “is associated with”) instead of causal language. We have also added explicit statements in the Limitations section clarifying that, given the cross-sectional design, the findings should be interpreted as theory-driven directional associations rather than empirically confirmed causal effects. The revised text appears in the manuscript (Lines 470-474).

“Second, the cross-sectional design precludes causal inference; although PLS-SEM facilitates the testing of theoretically specified directional relationships, it is essential to clarify that SEM methodologies do not prove causal assumptions; rather, they lend credibility to them [63]. Future longitudinal or experimental designs are needed to more rigorously validate these mechanisms.”

In addition, the Methods section (3.4 Statistical methods) was slightly refined to clarify the analytical procedure and to explicitly reference established PLS-SEM evaluation guidelines (Hair et al., 2017), without altering the analytical strategy. The revised text appears in the manuscript (Lines 324-330).

“The study used SmartPLS 4, partial least squares structural equation modeling. PLS-SEM was selected because it is well suited for models involving multiple latent constructs, does not require multivariate normality, and performs robustly with moderate sample sizes. These characteristics align with the analytical needs of the present study, which includes several psychological constructs measured with multi-item scales. Model evaluation followed established PLS-SEM guidelines [53], including assessments of measurement reliability, convergent validity, discriminant validity, and structural path estimates.”

5. The manuscript lists construct names but does not present the exact questionnaire items in the main text; the reader is directed to “Appendix A,” which is missing in the PDF. Include the full items or ensure Appendix A is attached, and clarify translation/validation procedures if the scales were adapted for a Taiwanese population.

Response:

We thank the reviewer for this important comment. Appendix A has been included and now contains the complete list of questionnaire items. In the revised manuscript, we added a new Appendix A to provide technical details of the VR system/modules. As a result, the questionnaire items have been moved to Appendix B, which now contains the complete list of items.

Because the study used established scales that required minor adaptation for the VR nursing context, we followed a standard translation and modification procedure. Items were translated into Chinese, checked by two bilingual researchers for equivalence, and reviewed by three experts in nursing education and VR training to confirm relevance and clarity before finalizing the wording. As referred to in the following paragraph in the manuscript:(See Line 317-322).

“Because the scales were adapted for use in a VR nursing-training context, all items underwent a structured translation process. Items were first translated into Chinese by a professional translation agency. Two bilingual researchers reviewed the wording for semantic equivalence and conceptual consistency. Three experts in nursing education and VR-based training assessed item relevance and contextual appropriateness, leading to minor refinements before finalization. Complete items are provided in Appendix B.”

Reviewer #2: Overall, the study shows that giving learners ways to actively interact with the system has a stronger impact than simply making the experience immersive. Interactivity boosts their confidence, motivation, and interest, and it also helps reduce unnecessary mental effort. The findings also show that when participants experienced visual discomfort—such as eye strain—it reduced both their sense of immersion and their willingness to interact. Interestingly, people who felt more neck, shoulder, or back pain actually reported feeling more immersed, possibly because they stayed in one posture for too long while deeply engaged. The study further notes that female participants tended to report more visual discomfort, which in turn lowered their immersion and interactivity, and younger participants were more sensitive to how visual discomfort affected their overall immersion.

We appreciate the reviewer’s thoughtful synthesis of the study’s key findings. In response, we have refined the manuscript to clarify theoretical interpretations, address causal limitations, and enhance the methodological clarity and practical implications of the study.

Main Review Questions
(These are high-level questions that address the core methodology, interpretation, and contributions of the study.

1.On the Counter-Intuitive Finding of NSB Pain and Immersion:

The positive association between neck, shoulder, and back (NSB) pain and immersion (β = 0.272, p < 0.05) is a highly unexpected and central finding. The discussion posits this may be due to heightened bodily awareness enhancing presence. Could the authors elaborate on this interpretation and discuss alternative explanations? For instance, could this correlation be influenced by unmeasured factors, such as the duration or intensity of physical engagement required by the tasks that might independently induce both NSB pain and a higher sense of immersion, making the relationship spurious?

Response:

Thank you for raising this important point. We appreciate the opportunity to clarify this unexpected finding and have substantially expanded the Discussion section to provide a more comprehensive interpretation. As referred to in the following paragraph in the manuscript:(See Line 436-448).

“A central and unexpected finding was the positive association between NSB pain and immersion. While earlier research has generally shown that musculoskeletal discomfort detracts from VR experience [60, 41], two explanations may account for the current pattern. First, procedural nursing tasks require sustained concentration and repeated upper-body movements. Learners who were more engaged may have simultaneously reported higher immersion and greater musculoskeletal strain. In such cases, NSB pain may reflect the bodily load associated with high task engagement rather than contributing directly to immersive experience. Second, prior research suggests that sensorimotor involvement and bodily feedback can shape users’ subjective experience in virtual environments [17]. From this perspective, musculoskeletal sensations may be integrated into the action–perception cycle during VR training, thereby co-occurring with heightened immersion. These interpretations remain associative rather than causal, but they highlight the nuanced ways in which physical sensations may accompany immersive learning experiences in action-oriented VR tasks.”

2.Reconciling Causal Hypotheses with a Cross-Sectional Design:
The study uses strong causal language in its hypotheses (e.g., "H1: Immersion with VR has positive impacts on...") and discussion, yet the cross-sectional design only allows for establishing correlations. A participant’s pre-existing high self-efficacy, for example, might cause them to perceive higher interactivity, rather than the other way around. How do the authors justify the causal framing of their findings, and could they explicitly address the limitations of inferring causality from their data in the discussion?

Response:

We appreciate this important clarification. We agree that the cross-sectional design cannot establish causality or rule out reverse associations (e.g., learners with higher pre-existing self-efficacy perceiving greater interactivity). In response, we have revised all hypotheses and interpretations to adopt associative language and added an explicit limitation noting that causal inference is not possible with the present design. The revised text appears in the manuscript (Lines 470–474):

“Second, the cross-sectional design precludes causal inference; although PLS-SEM facilitates the testing of theoretically specified directional relationships, it is essential to clarify that SEM methodologies do not prove causal assumptions; rather, they lend credibility to them [63]. Future longitudinal or experimental designs are needed to more rigorously validate these mechanisms.”

3.Investigating the Contradictory Finding on Interactivity and Cognitive Load: The finding that interactivity significantly *reduced* extraneous cognitive load (β = -0.566) directly contradicts hypothesis H2e and the theoretical basis suggesting that complex interactions can increase it. While the authors attribute this to well-designed tasks, this is a significant finding with major implications for instructional design. Could the authors elaborate on how "interactivity" in this specific application was conceptually distinct from the learning task itself? Is it possible that the implemented interactivity functioned as a form of instructional scaffolding (thus reducing cognitive load) rather than as a separate, potentially distracting feature?

Response:

Thank you for this insightful comment. We have revised the Discussion section to clarify why interactivity in our VR system was associated with reduced extraneous cognitive load. Specifically, we now explain that interactivity in this study was implemented as intuitive, low-complexity action pathways (controller manipulation and gaze-based selection) that were tightly integrated with instructional scaffolding, rather than introduced as additional task demands. The revised text appears in the manuscript (Lines418–423):

“…However, in this study, interactivity was implemented through intuitive and low-complexity action pathways embedded within the procedural sequences. Such design features may function as instructional scaffolding, guiding learners through the tasks and minimizing unnecessary processing demands, thus reducing extraneous load [59]. This finding underscores the importance of interface design in determining whether interactivity contributes to or alleviates cognitive burden.”

Minor Revision Questions
(These are more specific questions focused on clarity, completeness, and consistency, which are typically easier to address.)

1.Clarification of Exclusion Criteria:
Line 282 states that an exclusion criterion was "those who experienced discomfort during VR use." This appears to contradict the study's aim of measuring the effects of physical discomfort. Please clarify this criterion. Was it intended to exclude only participants who experienced debilitating discomfort that required them to withdraw from the training session?

Response:

Thank you for pointing out this ambiguity. We have revised the manuscript to reflect this clarification (Lines 269–272):

“The exclusion criteria included individuals with neuropsychiatric, cardiovascular, cognitive, and sensory disorders, as well as those who experienced severe or debilitating discomfort during VR use that prevented them from completing the session”

2.Completeness of the Path Model in Figure 2:

In Figure 2, the path diagram only presents the final significant paths. For completeness and transparency, please consider adding the non-significant paths that were tested (e.g., from Head Discomfort and Limb Pain to Immersion/Interactivity) as dotted lines with their corresponding non-significant coefficients. This would provide a more comprehensive overview of the full model that was analyzed.

Response:

Thank you for this helpful suggestion. In the revised manuscript, Figure 2 now clearly displays both significant and non-significant paths, with non-significant paths represented by dashed lines and accompanied by their corresponding coefficients. A legend has also been added to improve clarity and transparency. The updated Figure 2 has been included in the revised manuscript.

3.Rationale for the Age Cut-Off in Multi-Group Analysis:
The multi-group analysis uses a 35-year-old age cut-off, justified by citing Saredakis et al. [44]. Could the authors provide a brief rationale for why this specific cut-off is considered relevant for the current sample of nurse aide trainees? For instance, does this demarcation represent a meaningful split in terms of digital literacy, prior professional experience, or career stage within this cohort?

Response:

In the revised manuscript, we have clarified that the 35-year age cut-off was adopted to maintain consistency with prior VR research rather than based on assumptions about occupational stage or digital literacy. Specifically, Saredakis et al. (2020) identified this threshold as relevant when examining age-related differences in susceptibility to VR-induced discomfort. Because the present study focuses on physical discomfort in VR-based learning, adopting this established cut-off provided a theoretically grounded basis for the multi-group analysis. This clarification has been added to the manuscript (Lines 378–380).

“…This cutoff represents a theoretically meaningful midpoint, as prior research suggests that subtle age-related changes in vestibular and postural stability may begin to emerge around this period, which may in turn increase susceptibility to VR-induced physical discomfort [45].”

4.Operationalizing "Embodied Learning":
While Appendix A provides the questionnaire items for "Embodied Learning," the concept itself is complex. Could the authors add a brief operational definition in the Methods section (e.g., in 3.3 Measuring instrument) explaining what this construct is intended to capture in the specific context of procedural nursing skills in VR?

Response:

Thank you for this valuable suggestion. We have added a concise operational definition of embodied learning to the Methods section (Section 3.3) to clarify how this construct was conceptualized in the context of VR-based procedural nursing training. Specifically, embodied learning is defined as learners’ perceived extent to which their body movements and gestures support their understanding and performance of nursing procedures in VR. The revision has been incorporated into the manuscript (Lines 309–311).

“Embodied learning was operationalized as the extent to which learners perceived that their body movements and gestures supported their understanding and performance of the VR nursing procedures.”

5.Specificity of Practical Implications:

The conclusions urge educators to "consider these factors when planning VR courses." To make the findings more actionable, could the authors offer more concrete suggestions? For example, based on the results, should training sessions for younger or female participants be designed to be shorter, include more frequent breaks, or utilize different hardware settings to mitigate the greater impact of visual discomfort?

Response:

Thank you for this helpful suggestion. In the revised manuscript, we have added specific recommendations for VR course designers and educators, directly informed by the empirical findings.As referred to in the following paragraph in the manuscript:(See Line 458-465).

“Taken together, these results offer several practical implications. VR-based nursing education may benefit from incorporating adjustable hardware settings such as interpupillary distance, brightness control, and ergonomic headset configurations particularly for groups prone to visual discomfort. Shorter module durations or scheduled rest breaks may further reduce visual fatigue for younger or female learners [62,63]. Developers should continue to prioritize intuitive interaction design that supports task progression and minimizes extraneous load, and educators should select or design VR content with limited excessive motion to reduce the risk of discomfort.

”

6.The authors may consider citing and discussing the following related study, which presents a similar approach with your work. A. A. Laghari et al., “Quality of experience assessment in virtual/augmented reality serious games for healthcare: A systematic literature review,” Technology and Disability, vol. 36, no. 1–2, pp. 17–28, Feb. 2024, doi: 10.3233/tad-230035.

This paper could help strengthen the related work section and provide a clearer comparison with the proposed method.

Response:

Thank you for this valuable suggestion. We have incorporated Laghari et al. (2024) into multiple sections of the manuscript to better situate our study within the broader VR/AR healthcare literature. Specifically, this review is cited in Section 2.1 to highlight that prior work has largely focused on experiential aspects of VR, in Section 2.2 when discussing physical and visual discomfort as determinants of user engagement, and in the Discussion to emphasize how the present study extends this literature by linking discomfort factors to core learning mechanisms in VR-based nursing education.

These revisions appear in the manuscript (Lines 169-174;218-220;427-431).

“Recent systematic reviews of VR and AR applications in healthcare have highlighted immersion and interactivity as key determinants of users’ experience [27]. However, much of this work remains focused on experiential outcomes rather than on how these VR affordances relate to underlying learning mechanisms such as motivation, self-efficacy, or cognitive processing. Understanding these mechanisms is essential for explaining how learners engage with and interpret VR-based instructional environments”

“Prior VR/AR reviews in healthcare have also noted that visual and physical strain are important determinants of user experience, reflecting the physiological demands imposed by head-mounted displays and interactive environments [27]”

“VR/AR review studies in healthcare similarly emphasize visual strain as a leading determinant of reduced user experience [27]. The present findings extend this body of work by showing that visual discomfort is not merely detrimental at the experiential level but is also meaningfully associated with reduced immersive and interactive learning perceptions in procedural VR contexts.”

Laghari, A. A., Estrela, V. V., Li, H., Shoulin, Y., Khan, A. A., Anwar, M. S., ... & Bouraqia, K. (2024). Quality of experience assessment in virtual/augmented reality serious games for healthcare: A systematic literature review. *Technology and Disability*, *36*(1-2), 17-28.

Reviewer #3:

1.The manuscript is overly lengthy and would benefit from tighter organization—several sections contain redundant explanations that hinder readability
Response:

Thank you for this valuable suggestion. In response, we have substantially revised and streamlined the manuscript. Given the extent of the revisions, redundant explanations were removed, overlapping content across sections was consolidated, and the Introduction and Discussion were reorganized for clarity and conciseness. These revisions are reflected throughout the revised manuscript, particularly in the Introduction and Discussion sections, and can be clearly identified in the tracked-changes version provided. The revised manuscript was further professionally edited for clarity and conciseness.

2.Some hypotheses lack strong theoretical justification, especially those concerning physical discomfort; more prior literature should be cited to support these claims
Thank you for highlighting this issue.

We have strengthened the theoretical grounding of hypotheses related to physical discomfort by expanding the literature review in Section 2.2. Specifically, we incorporated additional references addressing visual discomfort, musculoskeletal strain, and VR-induced physical reactions, including recent reviews of VR user experience and discomfort in healthcare contexts. These additions provide clearer theoretical justification for the proposed associations between physical discomfort and immersive and interactive learning processes. The revised text appears in Section 2.2 (Lines 215–227).

“VR use can lead to various forms of physical discomfort, including visual discomfort, limb pain, neck–shoulder–back (NSB) pain, and head discomfort. These categories have been widely used in prior research to describe users’ physiological responses to immersive technology [18,40,41]. Prior VR/AR reviews in healthcare have also noted that visual and physical strain are important determinants of user experience, reflecting the physiological demands imposed by head-mounted displays and interactive environments [27]. Visual discomfort is frequently reported during VR use because head-mounted displays place sustained demands on the visual system, contributing to symptoms such as eyestrain or blurred vision [23]. Musculoskeletal pain may arise from repetitive or static postures maintained during VR interaction, particularly in the upper body [42, 43]. Head discomfort, including dizziness and headache, has been associated with visually induced motion sensations commonly described in cybersickness research [44, 45]. Although these reactions vary across individuals, they may influence users’ perceptual engagement and comfort during VR tasks.”

3.The discussion of unexpected findings (e.g., NSB pain increasing immersion) is speculative and needs stronger evidence or alternative explanations
Response:

Thank you for this important observation. We have substantially revised the Discussion section to clarify the interpretation of this unexpected finding and to avoid causal or speculative language. The revised discussion explicitly states that the observed association should not be interpreted as a causal effect. As referred to in the following paragraph in the manuscript:(See Line 436-448).

“A central and unexpected finding was the positive association between NSB pain and immersion. While earlier research has generally shown that musculoskeletal discomfort detracts from VR experience [60, 41], two explanations may account for the current pattern. First, procedural nursing tasks require sustained concentration and repeated upper-body movements. Learners who were more engaged may have simultaneously reported higher immersion and greater musculoskeletal strain. In such cases, NSB pain may reflect the bodily load associated with high task engagement rather than contributing directly to immersive experience. Second, prior research suggests that sensorimotor involvement and bodily feedback can shape users’ subjective experience in virtual environments [17]. From this perspective, musculoskeletal sensations may be integrated into the action–perception cycle during VR training, thereby co-occurring with heightened immersion. These interpretations remain associative rather than causal, but they highlight the nuanced ways in which physical sensations may accompany immersive learning experiences in action-oriented VR tasks.”

4.The multi-group analysis results are interesting, but the interpretation feels superficial—more depth is needed to explain gender and age differences
Response:

Thank you for this insightful observation. We have expanded the interpretation of the multi-group results to clarify why younger and female learners exhibited stronger effects of visual discomfort. The revised discussion appears in Lines449–465.

“The multi-group analyses further revealed meaningful subgroup differences. Female participants exhibited stronger negative associations between visual discomfort and immersion/interactivity than males, which accords with previous findings that interpupillary distance and visual fatigue may disproportionately affect women during VR use [44]. Younger learners experienced stronger negative associations between visual discomfort and immersion compared with older participants. This pattern may relate to greater cumulative exposure to digital screens among younger adults, which has been linked to higher susceptibility to ocular strain [62]. These findings underscore the importance of considering individual differences when designing and implementing VR-based instruction.

Taken together, these results offer several practical implications. VR-based nursing education may benefit from incorporating adjustable hardware settings such as interpupillary distance, brightness control, and ergonomic headset configurations particularly for groups prone to visual discomfort. Shorter module durations or scheduled rest breaks may further reduce visual fatigue for younger or female learners [62,63]. Developers should continue to prioritize intuitive interaction design that supports task progression and minimizes extraneous load, and educators should select or design VR content with limited excessive motion to reduce the risk of discomfort.

”

5.The study relies solely on self-reported questionnaires; the lack of objective physiological or behavioral measures limits the strength of the conclusions

Response:

Thank you for highlighting this methodological limitation. We have now clarified in the Limitations section that the study relied solely on self-reported measures, which may be subject to perceptual or recall biases. This statement has been added to the Limitations section (Lines 466–469).

“First, the study relied solely on self-reported measures, which may be subject to recall or response biases. Future research should incorporate objective indicators such as eye tracking, physiological measures, or behavioral performance data to provide a more comprehensive assessment of VR learning processes.”
